# Effects of Matrix Composition on Detection Threshold Estimates for Methyl Anthranilate and 2-Aminoacetophenone

**DOI:** 10.3390/foods5020035

**Published:** 2016-05-17

**Authors:** Demetra M. Perry, John E. Hayes

**Affiliations:** 1 Sensory Evaluation Center, College of Agricultural Sciences, the Pennsylvania State University, University Park, PA 16802 USA; dmp359@psu.edu; 2 Department of Food Science, College of Agricultural Sciences, the Pennslyvania State University, University Park, PA 16802 USA

**Keywords:** detection threshold, matrix effects, orthonasal olfaction, wine aroma, ascending forced-choice

## Abstract

Conceptually, a detection threshold represents the lowest concentration at which an individual or a group of individuals can reliably perceive a given stimulus, with a commonly used operational definition of 50% performance above chance. Estimated detection thresholds (DTs), however, are often reported in the literature with little attention given to the matrix in which the stimuli were evaluated. Here, we highlight the influence of matrix effects on DTs for two odor-active compounds commonly found in Vitis Labrusca wines. Differences in orthonasal DTs for methyl anthranilate (MA) and 2-aminoacetophenone (2AAP) in water, a model wine system, and wine were demonstrated using a within-subject design and forced choice (*i.e*., criterion free) psychophysical methods. Six sample triads, each containing two blanks and one spiked sample, were presented to participants with the instructions to choose the “different” sample, and this was repeated in different matrices (water, model wine, and wine). The estimated DTs for both compounds were significantly lower in water *versus* the model wine system and wine. This finding recapitulates the strong need to carefully consider the nature of the delivery matrix when determining and comparing threshold estimates across studies. Additionally, data from prior reports have suggested DTs for MA and 2AAP may differ by two orders of magnitude in spite of their structural similarity. We failed to confirm this difference here: although 2AAP thresholds were somewhat lower than MA thresholds, differences were much smaller than what had been suggested previously. This, again, emphasizes the need to make comparisons within the same individuals, using appropriate methods with sufficient numbers of participants.

## 1. Introduction

Aroma is an influential factor of flavor perception. Two distinct types of olfaction, typified by different routes of exposure, are known to influence sensory aspects of food products. These are orthonasal olfaction, where odorants reach olfactory receptors via the nostrils, and retronasal olfaction, where volatiles released during mastication and swallowing pass through the back of the throat to reach the same receptors. The concentrations of odorants that reach these receptors can range from levels well below threshold, where no sensation is perceived, to levels well in excess of threshold, where the sensation is clearly perceived. Within the flavor chemistry literature, the concentration of a specific odorant found within a food is often expressed in terms of an odor activity value (OAV), which is defined as the ratio of the concentration of the compound in the food over some estimate of the detection threshold for that compound.

Often, the literature reports detection thresholds for odor active compounds without emphasis on the matrix in which the value was estimated. This practice makes it difficult to evaluate the suitability of the threshold value for applications, such as the calculation of OAVs. This odor activity value should be assessed based on threshold estimates that were evaluated in the same matrix of interest for the OAV. For example, an air quality report listing OAVs for various odorants use threshold values where air is the diluent. Conversely, a fragrance company focused on product formulation would likely be more interested in threshold values based on assessments of the chemicals in water, water/alcohol matrices, or possibly oil. Thus, the choice of matrix is a critical consideration when reporting thresholds and determining OAVs to prevent widely varying and non-generalizable values from being perpetuated throughout the literature.

Beverages are complex systems, chemically and perceptually. Given the same concentration of a compound, it may be pronounced in one application and subtle in another. Potential masking effects have been studied with relation to aroma and taste perception, but the extent to which these effects may influence detection thresholds remains largely unanswered. To investigate differences in thresholds evaluated in diverse matrices, we used methyl anthranilate (MA) and 2-aminoacetophenone (2AAP), two odor-active compounds commonly found in *V. Labrusca* wines. Previous studies with 2AAP have suggested that detection thresholds may change with respect to the wine varietal in which the study is performed. These studies suggest a detection threshold for 2AAP of 0.5 μg/L in Pinot Gris and Chardonnay [1] and 2.0 μg/L in Riesling [2]. The more complex aroma profile of Riesling (e.g., floral, petrol) *versus* the simpler aroma profile of a typical Pinot Gris or Chardonnay suggests that perceptual masking effects may be an important part of understanding the underlying cause of matrix dependence of detection thresholds.

Prior reports also suggest there may be a two-log difference in detection thresholds for MA and 2AAP, despite their structural similarity. The detection threshold for MA in Riesling has been reported as 300 μg/L [3] as compared to an estimate of 2 μg/L for 2AAP in Riesling [2]. More recently, Czerny *et al*. [4] used a series of triangle tests with 13 to 22 participants to estimate detection thresholds for a wide range of compounds in water. They estimated the 2AAP detection threshold to be 0.27 μg/L in water. Critically, we were unable to find a direct comparison of the two compounds using the same group of participants in the same matrix. Consequently, a direct comparison of these compounds using a forced-choice procedure in a within-subjects design was performed here with a relatively large sample to elucidate potential differences in the detection thresholds for these compounds. This was done to mitigate differences that may have arisen in previous studies of the compounds from small numbers of participants and/or differences in psychophysical methods across studies.

Accordingly, the matrix dependency for detection thresholds was analyzed using methyl anthranilate and 2-aminoacetophenone as the volatile odor-active compounds of interest. Here, we investigated differences in threshold values as a function of the matrix, using water, model wine and wine as the matrices. Additionally, we explored the magnitude of the previously reported differences in detection thresholds between MA and 2AAP, two similarly structured compounds.

## 2. Materials and Methods

### 2.1. Overview

A total of five experiments were completed to study the effect of matrix on detection thresholds for MA and 2AAP. See Table 1 for a summary of the combinations of delivery matrices (water, model wine, and wine) and odorants (MA or 2AAP) that were compared. For example, in Experiment 2, we determined the detection thresholds for both MA and 2AAP in water using the same group of participants. For each experiment, the specific concentration series used in determining thresholds was adjusted as needed, depending on published literature and informal pilot testing. These details are provided below.

To estimate detection thresholds, a modified version of a standard triangle test was used. In a standard triangle test, the participant receives a set of three samples, two of which are the same, and one that is different, and they are tasked with identifying the sample that is different. This is constructed as a forced choice task, so participants must pick one as different even if they are forced to guess. The six possible presentation orders (AA’B, ABA’, BAA’, BB’A, BAB’, and ABB’) are counterbalanced across participants so half receive two of sample A and one of sample B while the other half receive one of sample A and two of sample B.

However, when used for threshold estimation, this paradigm is typically modified in one key respect. Instead of presenting half of participants with two control samples (vehicle only) and one spiked sample (vehicle + odorant), and half with one control sample and two spiked samples, all participants receive two control samples and one spiked sample, thereby reducing the possible number of presentation orders to three (AA’B, ABA’, and BAA’, or in this case CC’S, CSC’, and SCC’). This is done to minimize exposure to the odorant, to reduce the potential for adaptation. In any single triangle test, the vehicle was held constant, such that the odorant + vehicle was compared back to the same vehicle without any added odorant. Within each triad, the order of samples within each was counterbalanced across individuals.

For each compound and matrix, participants were presented with six separate sets (triads), and asked to evaluate them orthonasally (*i.e*., by sniffing without taking the sample into the mouth) and then identify one sample as being different. The triads were presented so that the spiked sample increased in concentration across the series, with the participant receiving the lowest spiked concentration for their first set (triad) of samples. Again, this was done to reduce adaptation effects, as participants never received a less concentrated spike after a more concentrated spike.

### 2.2. Stimuli

**Odorants**. Stock solutions were made using methyl anthranilate (99.5% purity, FG, Sigma-Aldrich, Milwaukee, WI, USA) and 2-aminoacetophenone (99.3% purity, FG, Sigma-Aldrich, Milwaukee, WI, USA). These were used “as is” from the supplier, and no attempt was made to purify them or to quantify trace impurities that may potentially be odor active.

**Wine**. Bulk Riesling (Franz Reinhart, Production Date July 10, 2014. Lot No. L-14191, Packed by Bingen am Rhein, Serial 4002301431350) was purchased locally from a retail store run by the Pennsylvania Liquor Control Board (PLCB).

**Model Wine**. A model wine system was created to match the pH, alcohol, and titratable acidity of the Franz Reinhart Riesling, which were measured using standard analytical techniques. The alcohol content of the Riesling was measured using ebulliometry [5]. Reverse osmosis water was used as the starting base for the model wine, and a sufficient volume of ethanol (190 proof, KOPTEC, King of Prussia, PA) was added to reach a final ethanol concentration of 9.65% *v/v*. The titratable acidity was adjusted to 8.15 g/L using tartaric acid (either 99.7% purity, FG, Sigma-Aldrich, Milwaukee, WI, USA; or 99.8% purity, FCC, Spectrum Chemicals, Gardena, CA, USA), consistent with Goodstein *et al*. [6], who modeled the titratable acidity of white wine using tartaric acid. The pH was adjusted to 3.10 using a solution of 5N sodium hydroxide (NaOH pellets, FCC, 97.8% purity, Macron Fine Chemicals, Sweden).

**Water**. Reverse osmosis (RO) water was used as the diluent for experiments where water was the delivery matrix.

### 2.3. Concentration Ranges

Within each experiment, participants received stimuli that covered 2.5 orders of magnitude in concentration, in half log steps. Details on the stimuli concentrations used in individual experiments are provided below.

Experiment 1. MA in Water *versus* Wine. Concentration series of 0.056, 0.18, 0.56, 1.8, 5.6, 18 μg/L in water and 0.18, 0.56, 1.8, 5.6, 18, 56 μg/L in wine were used to evaluate the detection threshold of MA.

Experiment 2. MA *versus* 2AAP in Water. Concentration series of MA 0.18, 0.56, 1.8, 5.6, 18, 56 μg/L of MA and 0.018, 0.056, 0.18, 0.56, 1.8, 5.6 μg/L of 2AAP were used to evaluate the detection thresholds of the two stimuli in water.

Experiment 3. 2AAP in Wine. A concentration series of 0.056, 0.18, 0.56, 1.8, 5.6, 18 μg/L was used to evaluate the detection threshold of 2AAP in wine.

Experiment 4. MA in Water *versus* Model Wine. Concentration series of 0.56, 1.8, 5.6, 18, 56, and 180 μg/L in water and 1.8, 5.6, 18, 56, 180, 560 μg/L in model wine were used to evaluate the detection threshold of MA.

Experiment 5. 2AAP in Water *versus* Model Wine. Concentration series of 0.056, 0.18, 0.56, 1.8, 5.6, 18 μg/L in water and 1.8, 5.6, 18, 56, 180, 560 μg/L in model wine were used to evaluate the detection threshold of 2AAP.

In all experiments, the required criterion for detection (detailed in Section 2.5.2 below) always fell within the range being tested for that odorant and matrix, suggesting the concentration series used were appropriate.

### 2.4. Sample Preparation

Solutions were stored in a refrigerator (34°F) until the day of the sensory test. Samples were prepared no more than seven days in advance of administering the sensory test. Solutions were removed from the refrigerator and allowed to come to room temperature before being given to participants.

### 2.5. Data Collection and Analysis

#### 2.5.1. Sensory Methodology

For determination of detection thresholds, participants received 30 mL samples served in standard ISO wine tasting glasses, each covered with a tightly fitting paper StanCap cover (Sonoco Paperboard Specialties, Norcross, GA, USA) labeled with a three digit blinding code. All glasses were prepared one hour before serving to allow for temperature and headspace equilibration. The samples were presented and evaluated in individual booths at room temperature (21 °C) under red light (100 W Sylvania 115–125V red flood light) in the Sensory Evaluation Center located in the Rodney E. Erickson Food Science Building at the Pennsylvania State University.

For each experiment, 48 participants were recruited from an existing pool of interested individuals who had previously opted-in to be contacted about taste and smell experiments in our facility. Following screening, scheduling was handled using software from SONA systems (Tallinn, Estonia). Healthy, non-smoking individuals with no known taste and smell defects who consumed white wine at least once every six months were invited to participate in the study. Individuals under the age of 21, as well as currently enrolled undergraduate students (regardless of age) were excluded from the study. The screening also asked individuals to indicate their consumption of real and fictitious wine varietals: participants who indicated they drank the fictitious varietal in the list (“Grebenheim”) were excluded from the study. All participants provided their informed consent before participating in the study. All study procedures were approved by The Pennsylvania State University Office for Research Protections (protocol number #44131).

With the exception of the experiment determining the detection threshold for 2AAP in wine (Experiment 3), all other experiments were two-day studies in which participants who completed day 1 of testing were asked to come back to complete the second part of the experiment within two days of initial testing in a crossover design. Sample presentation was counterbalanced such that half of the participants received one series of samples (e.g., MA) the first day and the second series (e.g., 2AAP) the second day while the other half received the second series the first day and the first series the second day. This was done to account for possible learning effects across repeated testing [7].

Participants were presented with six sets, containing three samples each, and asked to evaluate them based on orthonasal olfaction. Each set contained two samples of the control (untreated sample of water, model wine, or wine) and one sample of the diluent spiked with either 2AAP or MA. The triads were presented so that the spiked sample increased in concentration, with the subject receiving the lowest spiked concentration for their first set (triad) of samples. Within each triad, the order of samples within each was counterbalanced across individuals.

For each sample, participants were asked to swirl the glass three times before removing the paper cap, remove the cap, and smell the sample. Within the triad, they were told to identify which of the three was the “different” sample. Responses were collected electronically using Compusense five software (Compusense, Inc, Guelph, ON, Canada). Between each set of samples (each triad), subjects waited 90 s before proceeding, after which time they were presented with a screen instructing them to sample the next set. This 90-second break between triads was enforced via software. For additional details on sensory testing methods and best practices, the reader is referred to [7].

#### 2.5.2. Data Analysis

Data were exported from Compusense, sorted and tabulated at the end of each experiment. For the two-day studies, data were excluded for subjects that failed to return for the second day. Data were also excluded for non-responders, which we defined as subjects who were unable to complete the task successfully at least once (*i.e*., individuals who failed to correctly identify the different sample in at least one of the last three triads containing the three highest concentrations of stimulus). We calculated group detection thresholds graphically (e.g., [8]) based on sigmoidal fits of the group proportions of correct responses. We fit the threshold functions using the four parameter logistic nonlinear regression function (*i.e*., the Hill equation) in GraphPad Prism 5.0C for OSX (GraphPad Software, San Diego, CA, USA), which generated values for detection threshold, as well as 95% confidence intervals. In this analysis, detection threshold was defined as the concentration at which the resulting performance was 50% greater than chance [8]. That is, because the chance of guessing correctly in a triangle test is 1/3, and perfect performance is 1 (100% correct responses), a chance-adjusted proportion of 2/3 was used as the threshold criterion; this proportion was used to calculate the threshold concentration directly in software. As a secondary approach based on the ASTM E679 method, we also calculated individual best estimate thresholds (BETs) as the geometric mean of the highest concentration missed and the next highest concentration in which the different sample at all subsequent higher concentrations was selected correctly. Group BETs were calculated as the geometric means of the individual BETs [9]. These data were largely consistent with the regression approach using group proportions, and are reported in supplemental Table S1.

## 3. Results

### 3.1. Matrix Effects on Detection Thresholds

Classically, the detection threshold for a chemical is defined as the lowest concentration at which 50% of the population can identify its presence. While matrix effects are widely assumed to influence perception, there is a surprising paucity of work that systematically characterizes the extent to which the matrix affects the estimated detection threshold. Table 2 provides the detection thresholds for MA compiled from experiments 1, 2 and 4, which were determined graphically using a chance corrected method, similar to Lawless [8]. The values calculated using the group BET method outlined in the ASTM E-679 method were generally consistent with the values determined graphically (see Table S1).

Two results are clear from Table 2. First, with appropriate psychophysical methods, the detection thresholds for MA in water are consistent and reproducible across experiments, suggesting the methods used here to determine the detection threshold were robust. Second, the effect of the matrix is clearly demonstrated between water and wine, and water and model wine, as there are statistically significant differences for both comparisons, given that the confidence intervals do not overlap. Finally, we caution that the apparently higher value for model wine *versus* the wine should not be over interpreted, as the confidence intervals for these two matrices show substantial overlap.

**Figure 1:**
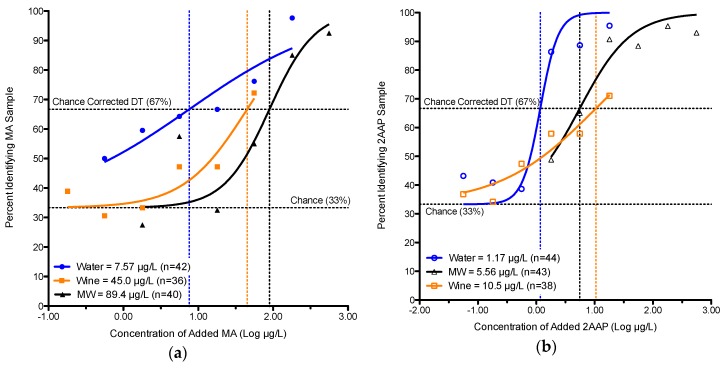
Detection Thresholds (DTs) across the three matrices. The sigmoidal curve fittings of group responses for the forced-choice tasks were calculated using the Hill equation. Separate fits are shown for: (**a**) methyl anthranilate and; (**b**) 2-aminoacetophenone in water (blue circle), model wine (black triangles) and wine (orange squares).

Table 3 summarizes the detection thresholds for 2AAP in water, model wine and wine. Experiment 5, a within-subjects design, showed a statistically significant difference (*p* < 0.05) in 2AAP detection thresholds in water *versus* model wine: the detection threshold for 2AAP in water was 1.17 μg/L compared to 5.56 μg/L for model wine. Also, a between-subjects comparison of water and wine detection thresholds for 2AAP (Experiment 2 *versus* 3) shows a statistically significant difference: 1.00 μg/L *versus* 10.5 μg/L. Table 4 summarizes the detection thresholds for both compounds across all matrices, with the sigmoidal fits for the evaluations shown in Figure 1.

### 3.2. Investigating Reported Differences in Detection Threshold between Methyl Anthranilate and 2-Aminoacetophenone

Extant literature fails to directly compare the detection threshold for MA to that of 2AAP using the same participants. To eliminate the possibility of confounding factors such as individual differences in perception, which is of particular concern for studies using small sample sizes, this study utilized a larger group of participants, based on a power calculation that suggested a minimum sample size of *n* = 22, to evaluate detection thresholds for both MA and 2AAP using a constant method.

**Figure 2:**
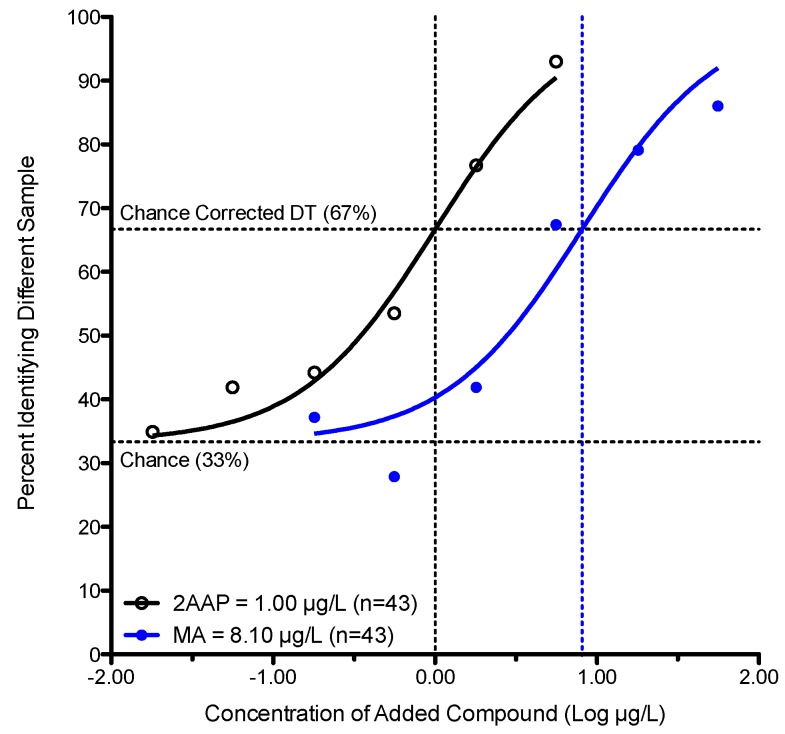
Detection Thresholds (DTs) for Methyl Anthranilate *versus* 2-aminoacetophenone in water. The group response rates were plotted against the log concentration of the added compound (2AAP: open black circles, MA: shaded blue circles) and fitted using the Hill Equation.

Existing literature reports a detection threshold for MA of 300 μg/L [3] and 0.5–2 μg/L for 2-aminoacetophenone [1,2,10] in various white wines, implying there is a two-log difference in the concentration required to reach the detection threshold. Here in Experiment 2, we used a within-subjects design to directly compare the detection threshold for MA *versus* 2AAP in water. The detection threshold for 2AAP was 1.0 μg/L (95% CI of 0.7–1.5 μg/L) compared to 8.1 μg/L for MA (95% CI of 3.8–17.2 μg/L). As Figure 2 shows, there is less than a 10 fold (one-log) difference in thresholds for these compounds, suggesting the ~100 fold (two-log) difference described previously may be an artifact of variable testing methods across studies and/or noise due to small sample sizes.

## 4. Discussion

### 4.1. Significance and Innovation

Previous studies estimating detection thresholds for MA and 2AAP have used ranking procedures with a few participants (>8) (e.g., [3]) or forced choice methods with slightly more participants (>22) (e.g., [4]). Notably, thresholds for these two compounds have never been estimated in the same group of participants. Here, we used the forced-choice methodology outlined in ASTM method E-679 with a larger group of participants (*n*’s of 36 to 44). Notably, the E-679 method differs slightly from both standard 3-AFC tests (which asks participants to identify the strongest or weakest sample) and the triangle test (which asks participants to identify the different sample). Moreover, a standard triangle test would typically be counterbalanced, such that half of participants receive two of sample A and one of sample B, while the other half of participants receive two of sample B and one of sample A. The ASTM E-679 method for threshold determination intentionally blends these two methods: as in a 3-AFC test, participants always receive one spike (vehicle + odorant) and two blanks (vehicle only). This is done to minimize exposure to the odorant to reduce potential for adaptation. However, as in a triangle test, the participants are instructed to pick the different sample, thereby requiring a different cognitive strategy on the part of the participant (differencing *versus* skimming). Also, the primary objective of this study was to estimate detection thresholds as they might occur in the general population across a series of different delivery matrices; therefore untrained panelists (*n*’s = 35 to 44) were used to evaluate detection threshold, rather than trained panelists. These methodological differences may explain the deviation of our estimates from previously published values regarding the detection threshold for MA in wine. The stability and consistency of our results across multiple experiments reported here suggests the present values may be more accurate than previously published values for these two compounds.

In this study, we also found the detection threshold concentrations for MA and 2AAP to be highly matrix dependent. This is consistent with the findings by Martineau *et al*. [11] whose study noted differences in detection threshold values for diacetyl as a function of wine variety. Additionally, we found that the differences between MA and 2AAP were not as great as might be expected from different reports in prior literature. We speculate this was due to the use of larger numbers of participants here as well as use of a consistent psychophysical method (a forced-choice task) for MA and 2AAP across all matrices. Accordingly, variability that would normally arise as a consequence of methodological variation between studies, as well as innate individual differences across participants, was substantially reduced. Finally, we propose use of sigmoidal fits to model the participant responses, while also using the chance-corrected factor described elsewhere by Lawless [8], may be a more appropriate and robust method that should be used in future studies. Critically, threshold values are only ever estimates based on probabilities. However, these estimates are typically assumed to be highly precise, when they are not: most publications never bother to calculate or report confidence intervals for their threshold estimates. By coupling a standard model from pharmacology (the Hill equation) along with a classical definition of a threshold (50% of the population, adjusted for chance), we are able to easily calculate confidence intervals. If more researchers did so, this would reinforce the probabilistic nature of these estimates. Finally, given the matrix dependency shown here, this work recapitulates the need to use an appropriate delivery system for psychophysical studies on aroma perception, as well as flavor chemistry studies that express concentrations in terms of odor-activity values.

### 4.2. Study Limitations and Future Directions

A model wine system was included here as an attempt to differentiate perceptual masking effects from physical partitioning effects as a potential mechanism to explain the observation that detection thresholds for both compounds were consistently higher in wine than in water. However, ethanol, a key component of model wine, is also odor active at the concentrations used here [12,13], so it remains unknown whether differences in detection threshold result as an effect of perceptual masking or partitioning phenomena. Given this, it is not possible to directly answer the question of masking *versus* partitioning using solely psychophysical testing, as we cannot deconfound the odor activity from ethanol from its role in partitioning. That said, we did not find any evidence suggesting that DTs were higher in wine than in model wine, which might be anticipated due to the presence of many additional odor active chemicals in real wine [14,15]. Tentatively then, this suggests either (a) the shift in threshold is due to simple partitioning, or (b) that the olfactory sensations from ethanol alone are sufficient to cause this shift (*i.e.*, additional odorants are not required to shift the thresholds for 2AAP and MA upward).

Also, this study focused solely on orthonasal detection thresholds. It is unknown how this work may translate to retronasal delivery of these odorants, as saliva [16], warming and agitation in the mouth may also influence partitioning [17]. Additional considerations would include the role of cross modal mixture suppression from chemesthetic or tactile input (e.g., ethanol burn or viscosity changes).

## 5. Conclusions

Data presented here show detection thresholds for MA and 2AAP were significantly lower in water *versus* model wine or wine, recapitulating the strong need to carefully consider the composition of the delivery matrix when determining and comparing threshold estimates across studies. Additionally, we found detection thresholds to be quite stable across experiments when appropriate methods with sufficient numbers of participants were used. Finally, we found evidence that prior studies may have overstated the relative difference in detection thresholds for these two compounds: while 2AAP thresholds were somewhat lower than MA thresholds, the differences observed were much smaller than suggested previously.

## Figures and Tables

**Table 1:** Overview of five experiments, summarizing the relative comparisons of which odorants were tested, as well as the matrix they were tested in, for each experiment.

	Water	Model Wine	Wine
Experiment 1	MA		MA
Experiment 2	MA *vs*. 2AAP		
Experiment 3			2AAP
Experiment 4	MA	MA	
Experiment 5	2AAP	2AAP	

**Table 2:** Detection Thresholds (μg/L) for Methyl Anthranilate.

	Water			Wine			Model Wine		
	DT	95% CI	*n* *	DT	95% CI	*n*	DT	95% CI	*n*
Experiment 1	7.51	3.1–17.9	38	45.0	21.2–95.5	36	-	-	-
Experiment 2	8.10	3.82–17.2	43	-	-	-	-	-	-
Experiment 4	7.57	2.30–25.0	42	-	-	-	89.4	28.2–283	40

* *n* indicates sample size.

**Table 3:** Detection Thresholds (μg/L) for 2-aminoacetophenone.

	Water			Wine			Model Wine		
	DT	95% CI	*n* *	DT	95% CI	*n*	DT	95% CI	*n*
Experiment 2	1.00	0.685–1.47	43	-	-		-	-	
Experiment 3	-	-	-	10.5	3.79–29.3	38	-	-	
Experiment 5	1.17	0.614–2.24	44	-	-		5.56	2.94–10.5	43

* *n* indicates sample size.

**Table 4:** Mean Detection Thresholds (μg/L) across All Matrices.

	Water ^1^		Wine		Model Wine	
	DT	95% CI	DT	95% CI	DT	95% CI
MA	7.73	-	45.0	21.2–95.5	89.4	28.2–283
2AAP	1.09	-	10.5	3.79–29.3	5.56	2.94–10.5

^1^ Values reported here were determined by calculating the mean of values generated from individual sigmoidal fits across the relevant experiments.

## Supplementary Materials

The following are available online at www.mdpi.com/2304-8158/5/2/35/s1, Table S1: Group BETs (μg/L) and Graphically Determined Detection Thresholds (μg/L).

## Abbreviations

The following abbreviations are used in this manuscript:

MA: methyl anthranilate
2AAP: 2-aminoacetophenone
OAV: odor activity value
DT: detection threshold
CT: confidence interval
