# Supplementary material for: Effects of Matrix Composition on Detection Threshold Estimates for Methyl Anthranilate and 2-Aminoacetophenone
Source: Foods. 2016 May 17;5(2):35. doi: 10.3390/foods5020035 (PMC5302346; doi:10.3390/foods5020035)
Supplement: Supplementary file 1 [file foods-05-00035-s001.docx]

Effects of Matrix Composition on Detection Threshold Estimates for Methyl Anthranilate and 2-Aminoacetophenone

Demetra M. Perry and John E. Hayes

Group Best Estimate Thresholds; Comparison to Graphical Calculations

Group best estimate thresholds (BETs) were calculated for each experiment based on individual BETs. Individual BETs were calculated as the geometric mean of the highest concentration missed in the 3-AFC tasks and the next concentration in the ascending series that was chosen correctly and followed by at least three subsequent correct responses, or all remaining in the series.

The group BETs were based on individual BETs, which were determined from one trial. The experiments would need to be repeated to collect a standard deviation for individual BETs. The group BETs and 95% CI are combined with the detection thresholds determined graphically, which is to say the concentration of added compounds at which 50% of respondents perform greater than chance (67% correctly identify the “different” sample).

**Table S1.** Group BETs (μg/L) and Graphically Determined Detection Thresholds (μg/L).

| **Experiment 1** | **Water** | | | | **Wine** | | | | |  |
| --- | --- | --- | --- | --- | --- | --- | --- | --- | --- | --- |
|  | Group BET | | Graph | | | Group BET | | | Graph | |
| MA | 3.924 | | 7.514 | | | 20.29 | | | 44.98 | |
| 95% CI | 2.339, 6.581 | | 3.148, 17.93 | | | 12. 46, 33.05 | | | 21.18, 95.54 | |
| **Experiment 2** | **Water** | | | |  | | | | |  |
|  | Group BET | | Graph | |  | | |  | |  |
| MA | 5.722 | | 8.097 | |  | | |  | |  |
| 95% CI | 3.184, 10.28 | | 3.824, 17.15 | |  | | |  | |  |
| 2AAP | 0.5722 | | 1.002 | |  | | |  | |  |
| 95% CI | 0.3433, 0.9536 | | 0.6852, 1.465 | |  | | |  | |  |
| **Experiment 3** | **Water** | | | | **Wine** | | | | |  |
|  |  | | | | Group BET | | | Graph | |  |
| 2AAP |  | | | | 4.431 | | | 10.54 | |  |
| 95% CI |  | | | | 2.556, 7.680 | | | 3.791, 29.30 | |  |
| **Experiment 4** | **Water** | | | | **Model Wine** | | | | |  |
|  | Group BET | Graph | | Group BET | | | Graph | | |  |
| MA | 7.633 | 7.572 | | 50.32 | | | 89.36 | | |  |
| 95% CI | 4.140, 14.07 | 2.296, 24.97 | | 28.82, 87.85 | | | 28.19, 283.3 | | |  |
| **Experiment 5** | **Water** | | | | **Model Wine** | | | | |  |
|  | Group BET | Graph | | Group BET | | | Graph | | |  |
| 2AAP | 0.814 | 1.172 | | 5.28 | | | 5.564 | | |  |
| 95% CI | 0.5230, 1.268 | 0.6137, 2.238 | | 3.056, 9.124 | | | 2.942, 10.53 | | |  |
